# Transforming Growth Factor-β1 Signaling Represses Testicular Steroidogenesis through Cross-Talk with Orphan Nuclear Receptor Nur77

**DOI:** 10.1371/journal.pone.0104812

**Published:** 2014-08-20

**Authors:** Eunsook Park, Chin-Hee Song, Jae-Il Park, Ryun-Sup Ahn, Hueng-Sik Choi, CheMyong Ko, Keesook Lee

**Affiliations:** 1 Hormone Research Center, School of Biological Sciences and Technology, Chonnam National University, Gwangju, Republic of Korea; 2 Korea Basic Science Institute, Gwangju Center at Chonnam National University, Gwangju, Republic of Korea; 3 Graduate School of Integrative Medicine, CHA Medical University, Seoul, Republic of Korea; 4 Department of Comparative Biosciences, College of Veterinary Medicine, University of Illinois at Urbana-Champaign, Urbana, Illinois, United States of America; Georgia Regents University, United States of America

## Abstract

Transforming growth factor- β1 (TGF-β1) has been reported to inhibit luteinizing hormone (LH) mediated-steroidogenesis in testicular Leydig cells. However, the mechanism by which TGF-β1 controls the steroidogenesis in Leydig cells is not well understood. Here, we investigated the possibility that TGF-β1 represses steroidogenesis through cross-talk with the orphan nuclear receptor Nur77. Nur77, which is induced by LH/cAMP signaling, is one of major transcription factors that regulate the expression of steroidogenic genes in Leydig cells. TGF-β1 signaling inhibited cAMP-induced testosterone production and the expression of steroidogenic genes such as P450c17, StAR and 3β-HSD in mouse Leydig cells. Further, TGF-β1/ALK5 signaling repressed cAMP-induced and Nur77-activated promoter activity of steroidogenic genes. In addition, TGF-β1/ALK5-activated Smad3 repressed Nur77 transactivation of steroidogenic gene promoters by interfering with Nur77 binding to DNA. In primary Leydig cells isolated from Tgfbr2^flox/flox^ Cyp17iCre mice, TGF-β1-mediated repression of cAMP-induced steroidogenic gene expression was significantly less than that in primary Leydig cells from Tgfbr2^flox/flox^ mice. Taken together, these results suggest that TGF-β1/ALK5/Smad3 signaling represses the expression of steroidogenic genes via the suppression of Nur77 transactivation in testicular Leydig cells. These findings may provide a molecular mechanism involved in the TGF-β1-mediated repression of testicular steroidogenesis.

## Introduction

Steroidogenesis, the process of testosterone production, in testicular Leydig cells is controlled by luteinizing hormone (LH), which is synthesized and secreted from the pituitary. The intracellular second messenger for LH, cAMP, stimulates steroidogenesis by increasing the expression of several steroidogenic genes, including steroidogenic acute regulatory protein (StAR), cholesterol side chain cleavage cytochrome P450 (P450scc), 3β-hydroxysteroid dehydrogenase/isomerase (3β-HSD) and cytochrome P450 17α-hydroxylase/C_17–20_ lyase (P450c17) [1]. Steroidogenesis in Leydig cells is initiated by the translocation of cholesterol from the outer to the inner mitochondrial membrane, which is mediated by StAR. In the inner mitochondrial membrane, cholesterol is converted to pregnenolone by P450scc. Pregnenolone is then transported to the smooth endoplasmic reticulum and is converted to testosterone by a series of enzymes, including 3β-HSD and P450c17 [1].

The expression of steroidogenic genes is regulated by various transcription factors [2]. The orphan nuclear receptor Nur77 (also known as NR4A1, NGFI-B, TR3, and NAK-1) is one of the major transcription factors involved in the regulation of steroidogenic gene expression in Leydig cells [2], [3]. Like other nuclear receptors, Nur77 contains three functional domains: the N-terminal AF-1 domain, the DNA binding domain, and the C-terminal ligand binding domain containing another transactivation domain, AF-2 [4], [5]. Nur77 binds as monomer to the NGF1-B response element (NBRE) and as a homodimer or heterodimer to the Nur response element (NurRE) [6], [7]. Previous studies demonstrated that LH, the regulator of testicular steroidogenesis, induces Nur77 gene expression in Leydig cells [8] and that Nur77 regulates the expression of steroidogenic genes, including steroid 21-hydroxylase, 20α-hydroxysteroid dehydrogenase, and P450c17 [2], [9], [10]. Furthermore, Nur77-binding regions have been defined within the promoters of rat P450c17 [2], mouse StAR [11], and human 3β-HSD type 2 (3β-HSD2) [12] genes.

TGF-β, a member of the transforming growth factor-β (TGF-β) superfamily, regulates cell cycle progression and differentiation in a broad range of tissues under normal and pathological conditions [13], [14]. In the testis, TGF-β regulates a variety of cellular processes, including the secretory function of Leydig and Sertoli cells, as well as the organization of peritubular myoid cells, testis development and spermatogenesis [15], [16]. TGF-β signaling occurs through TGF-β type II receptor (TGF-βRII) and TGF-β type I receptor (TGF-βRI), also termed activin receptor-like kinase-5 (ALK5), both of which are serine/threonine kinase receptors. Binding of TGF-β to TGF-βRII induces the formation of hetromeric complexes with ALK5, within which TGF-βRII phosphorylates ALK5, turning on receptor kinase activity. The activated ALK5 subsequently induces Smad2 and/or Smad3 phosphorylation at C-terminal serines. Activated Smad2 and/or Smad3 form a heterotrimeric complex with Smad4, which then translocates to the nucleus. In the nucleus, Smad interacts with transcription factors at the promoter of TGF-β responsive genes to regulate transcription [17]–[19].

TGF-β1 has been shown to regulate the function of testicular Leydig cells *in vivo* and *in vitro*. TGF-β1 is secreted by porcine and rat Leydig cells, and its expression is regulated during the developmental stage of postnatal Leydig cells [20]–[22]. It represses hCG-induced testosterone production in Leydig cells through decreasing LH/hCG receptor expression and the expression of steroidogenic genes such as StAR and P450c17 [23]. TGF-β1 null mutant mice that survive to reproductive age have reduced testicular and serum testosterone levels, which is secondary to the deficiency of circulating LH [24]. It has also been reported that TGF-β1 inhibits cAMP-induced testosterone formation in primary Leydig cells [25]. Because cAMP is the intracellular messenger of LH signaling, it is possible that TGF-β1 signaling may also inhibit steroidogenesis through directly regulating the expression of steroidogenic genes that are induced by cAMP.

In the present study, we demonstrate that the inhibitory effect of TGF-β1 on testicular steroidogenesis occurs, at least in part, through the cross-talk of TGF-β1/ALK5-activated Smad3 with orphan nuclear receptor Nur77, and thus, Smad3 indirectly regulates the promoter activity of steroidogenic genes. These findings may provide a molecular mechanism for TGF-β1-mediated repression of testosterone production in testicular Leydig cells.

## Materials and Methods

### Plasmids and Chemicals

The mammalian expression vector for Nur77, pcDNA3HA-Nur77, and the reporter plasmids NurRE-luc and NBRE-luc were previously described [26]. Bacterial expression vectors of glutathione S-transferase (GST)-Nur77 and GST-Nur77 domain mutants, mouse StAR(−2200/+3)-Luc, mouse P450c17(−1040)-Luc, WT(−447/−399) P450c17-Luc, Mut(−447/−399Δ2) P450c17-Luc, and mouse 3β-HSD-Luc were also previously described [2], [26]. The pcDNA3HA-ALK5 mutant (WT, TD and KR) plasmids were previously described [27]. CS2-Flag-Smad3 and Flag-Smad3 phosphorylation mutants (S3A; S^422^SVS^425^-A^422^AVA^425^ and S3D; S^422^SVS^425^-D^422^DVD^425^) were previously described [28], [29]. The pcDNA3HA-Smad3 was constructed by inserting an EcoRI-SalI-digested Smad3 fragment from GST-Smad3 [30] into EcoRI-XhoI-digested pcDNA3HA. phmKG_N-MC-NLS-Smad3 was constructed by inserting a KpnI-XbaI-digested NLS-Smad3 fragment from pcDNA3HA-NLS-Smad3 into KpnI-XbaI-digested phmKG_N-MC. phmKG_C-MC-Nur77 and phmKG_C-MN-Nur77 were constructed by inserting a BamHI-HindIII-digested Nur77 fragment from pcDNA3HA-Nur77 into BamHI-HindIII-digested phmKG_C-MC and phmKG_C-MN, respectively.

Recombinant human TGF-β1 was purchased from Humanzyme (Chicago, IL). 8-bromo-cAMP (8Br-cAMP) and SB431542 were purchased from Sigma-Aldrich (St. Louis, MO).

### Cell culture, transfection and reporter assays

Mouse Leydig tumor MA-10 cells were kindly provided by Dr. M. Ascoli (University of Iowa, Iowa) and maintained in RPMI 1640 medium (HyClone, Logan, Utah) supplemented with 15% horse serum (Gibco, Carlsbad, CA) and antibiotics [31]. Rat Leydig tumor R2C cells were purchased from ATCC (Manassas, VA) and maintained in F10 medium supplemented with 15% horse serum, 2.5% fetal bovine serum (FBS, HyClone) and antibiotics. HeLa and HEK293T cells were maintained in Dulbecco’s minimum essential medium (DMEM) (HyClone) supplemented with 10% FBS and antibiotics. The cells were cultured at 37°C under an atmosphere of 5% CO_2_.

Cell transfections were performed using Lipofectamine 2000 transfection reagent (Invitrogen, Carlsbad, CA) according to the manufacturer’s instructions. For the luciferase reporter assay, cells were plated in medium containing 5% charcoal-stripped FBS for 24 hours prior to transfection. Cells were transfected with expression vectors, a reporter gene, and the control *lacZ* expression plasmid, pCMVβ (Clontech, Palo Alto, CA) or pSV-β-gal (Promega, Madison, WI). Cells were lysed with lysis buffer containing 0.1% Triton X-100 and 0.2 M Tris-HCl (pH 8.0). Luciferase and β-galactosidase activities were assayed as described previously [26]. The levels of luciferase activity were normalized to *lacZ* expression.

### Preparation of primary leydig cells

Preparation of mouse Leydig cells was carried out as previously described [31]. Briefly, the mice at 12 weeks were sacrificed by cervical dislocation and testes were collected. Testicular cells were dispersed by treating the decapsulated testes with collagenase type I (0.25 mg/ml, Sigma-Aldrich). The dispersed tissues were filtered with a 40-mm cell strainer (BD Biosciences, San Diego, CA) and interstitial cells were precipitated by centrifugation of the filtrate. Enrichment for Leydig cells was estimated by 3β-HSD immunocytochemistry, and the population of Leydig cells was 60–70% of total purified cells.

### Quantitative real-time polymerase chain reaction (qRT-PCR) and reverse transcriptase-polymerase chain reaction (RT-PCR) analysis

Total RNAs were prepared by using Tri-Reagent (Molecular Research Center, Inc., Cincinnati, OH) according to the manufacturer’s instructions. Two µg of total RNA isolated from cells was used for reverse transcription (RT) with M-MLV RT (Promega). Quantitative real-time PCR was performed using the StepOnePlus Real-Time PCR System (Applied Biosystems, Carlsbad, CA) and the SensiMixPlus SYBR Kit (Quantace, London, UK) according to the manufacturer’s procedure. The primer sequences for the genes were as follows: P450c17-F: 5′-CCAGGACCCAAGTGTGTTCT-3′; P450c17-R: 5′-CCTGATACGAAGCACTTCTCG-3′; StAR-F: 5′-TGTCAAGGAGATCAAGGTCCTG-3′; StAR-R: 5′-CGATAGGACCTGGTTGATGAT-3′; 3β-HSD-F: 5′-ATGGTCTGCCTGGGAATGAC-3′; 3β-HSD-R: 5′-ACTGCAGGAGGTCAGAGCT-3′ [32]; Tgfbr2-F: 5′-TGCAATGCTGTGGGAGAA-3′; Tgfbr2-R: 5′-GATCTGGATGCCCTGGTG-3′; Tgfbr1-F: 5′-CACCGTGTGCCAAATGAA-3′; Tgfbr1-R: 5′-TGCCTCGCCAAACTTCTC-3′. Finally, mRNA levels were normalized to β-actin (actin-F: 5′-GAGACCTTCAACACCCCAGCC-3′; actin-R: 5′-CCGTCAGGCAGCTCATAGCTC-3′).

### Western blot analysis

Western blot analysis was conducted as previously described [31]. The nuclear/cytosol fractionation kit (Bio Vision Technology Inc., Canada) was used to separate nuclear and cytoplasmic proteins, according to the manufacturer's protocol. Proteins were separated by sodium dodecyl sulfate-polyacrylamide gel electrophoresis (SDS-PAGE) and transferred to Protran nitrocellulose membranes (Whatman GmbH, Dassel, Germany). The signals were then detected with an Amersham ECL kit (GE Healthcare, Buckinghamshire, UK) and exposed to Amersham Hyperfilm ECL (GE Healthcare). The following antibodies were used: anti-Flag, (Sigma) anti-Smad3 [pSpS^423/425^], anti-Smad2/3 (Invitrogen, Carlsbad, CA), anti-GAPDH (Epitomics, Burlingame, CA), anti-P450c17, anti-StAR, anti-3β-HSD, anti-Nur77, anti-β-gal, anti-α-Tubulin and anti-Lamin B (Santa Cruz Biotechnology, Santa Cruz, CA).

### GST Pull-down assay

GST, GST-Nur77, and GST-Nur77 deleted mutant fusion proteins were expressed in *Escherichia coli* BL21 cells and isolated with glutathione-Sepharose-4B beads (GE Healthcare). The immobilized GST fusion proteins were then incubated with [^35^S] methionine-labeled proteins produced by *in vitro* translation using the TNT-coupled transcription-translation system (Promega). The binding reactions were performed in 400 µl of GST binding buffer (20 mM Tris (pH 7.9), 150 mM NaCl, 10% glycerol, 0.05% NP-40, 5 mM MgCl_2_, 0.5 mM EDTA, 1 mM dithiothreitol and 1.5% bovine serum albumin) overnight at 4°C. The beads were washed four times with GST binding buffer containing protease inhibitors. Bound proteins were analyzed by SDS-PAGE and autoradiography [33].

### Protein fragment complementation analysis (FCA)

To visualize the in vivo interaction, fluorescence protein fragment complementation methods with the Fluo-Chase kit (MBL International Corporation, Woburn, MA) were utilized according to the manufacturer’s procedure. As recommended, combinational pairs of mKG_N- and mKG_C-chimeric protein constructs were transfected into HeLa cells and fluorescence was visualized at 24 hours post-transfection using a Leica TCS SP5 AOBS laser scanning confocal microscope (Leica Microsystems) with a 63x oil objective. Fluorescence images were acquired as confocal stacks of 6–10 optical sections of 512×512 pixels and were averaged four times to reduce noise.

### siRNA experiment

Smad2, Smad3, Nur77 and scrambled small interfering RNAs (siRNAs) were ordered from Bioneer Technology (Daejon, Republic of Korea). The sense sequence for the siRNAs was as follows: Smad2 siRNA: 5′-GCAGAUUUUCCUUGUAGAAdTdT-3′; Smad3 siRNA: 5′-GCGUAUAGGUGAUGUACAGdTdT-3′; Nur77 siRNA: 5′-UCCCUGGCUUCAUUGAGCUUdTdT-3′; scrambled siRNA: 5′-CCUACGCCACCAAUUUGGUdTdT-3′. MA-10 and HEK293T cells were transfected with 20 nM siRNA using Lipofectamine 2000 (Invitrogen) according to the manufacturer’s instructions.

### Electrophoretic mobility shift assay (EMSA)

The GST fusion proteins (GST, GST-Nur77 and GST-Smad3) were expressed from *Escherichia coli* BL21 cells and purified with glutathione-Sepharose-4B beads. The NBRE oligonucleotide (5′-GGGGCTCGTGCGAAAAGGTCAAGCGCTA-3′) was annealed to its complementary oligonucleotide to form a double-strand, which was labeled with [α-^32^P] dCTP and purified using Sephadex G50 spin columns. EMSA was performed according to previously described procedures [34].

### Chromatin immunoprecipitation (ChIP) assay

Purified primary Leydig and R2C cells were treated with the indicated amounts of reagents for the indicated times and cross-linked with 1% formaldehyde. The cells were then processed for ChIP assays as previously described [31]. Anti-Nur77 antibody (sc-5569, Santa Cruz Biotechnology) was used for immunoprecipitation. The immunoprecipitated DNA and the input-sheared DNA were subjected to PCR using a primer pair for the mouse or rat P450c17 promoter, which amplifies the proximal region containing the Nur77 binding site [31]. As a negative control, PCR reactions were performed using a GAPDH primer pair, which amplifies the coding region of the GAPDH gene.

### Radioimmunoassay (RIA)

Testes from male mice or media from cultures of primary Leydig cells and R2C cells were prepared. Dissected testes were homogenized in phosphate-buffered saline (0.01 M; pH 7.2), and the steroids were extracted with diethyl ether. Testosterone concentrations were measured by radioimmunoassay, as described previously [31].

### Ethics Statement

All animal procedures were approved by the Institutional Animal Care and Use Committee (IACUC) of Chonnam National University (Permit Number: 2012-44).

### Animals

Leydig cell-specific TGF-β type II receptor (Tgfbr2) conditional knock-out (Tgfbr2^flox/flox^ Cyp17iCre) mice were obtained by crossing with Tgfbr2^flox/flox^ mice [35] and Cyp17iCre T/+ mice [36]. Tgfbr2^flox/flox^ mice, which contain two loxP sites in the introns flanking exon 2 of TGF-β type II receptor (Tgfbr2) gene, were used as a target for Tgfbr2 gene excision. Cyp17iCre T/+ mice, which express the Cre recombinase (iCre) gene under regulation of the Cyp17 promoter, were used to selectively delete Tgfbr2 in Leydig cells. Tgfbr2^flox/flox^ female mice were first crossed with Cyp17iCre T/+ male mice. The F1 heterozygote (Tgfbr2^fl^°^x/+^ Cyp17iCre T/+) male mice were then bred with Tgfbr2^flox/flox^ female mice, which gave the Tgfbr2^flox/flox^ Cyp17iCre T/+ and Tgfbr2^flox/flox^ genotypes.

### Statistical analysis

All cell results are presented as the standard error of the mean (SEM), and the animal data are presented as the standard deviation (SD) of at least three independent experiments. Statistical significance was calculated by two-tailed unpaired Student’s *t* test using the GraphPad Prism5 software. For all statistical analyses, *P<0.05* was considered significant.

## Results

### TGF-β1/ALK5 signaling represses cAMP-induced promoter activity of steroidogenic genes in testicular Leydig cells

**Figure 1:**
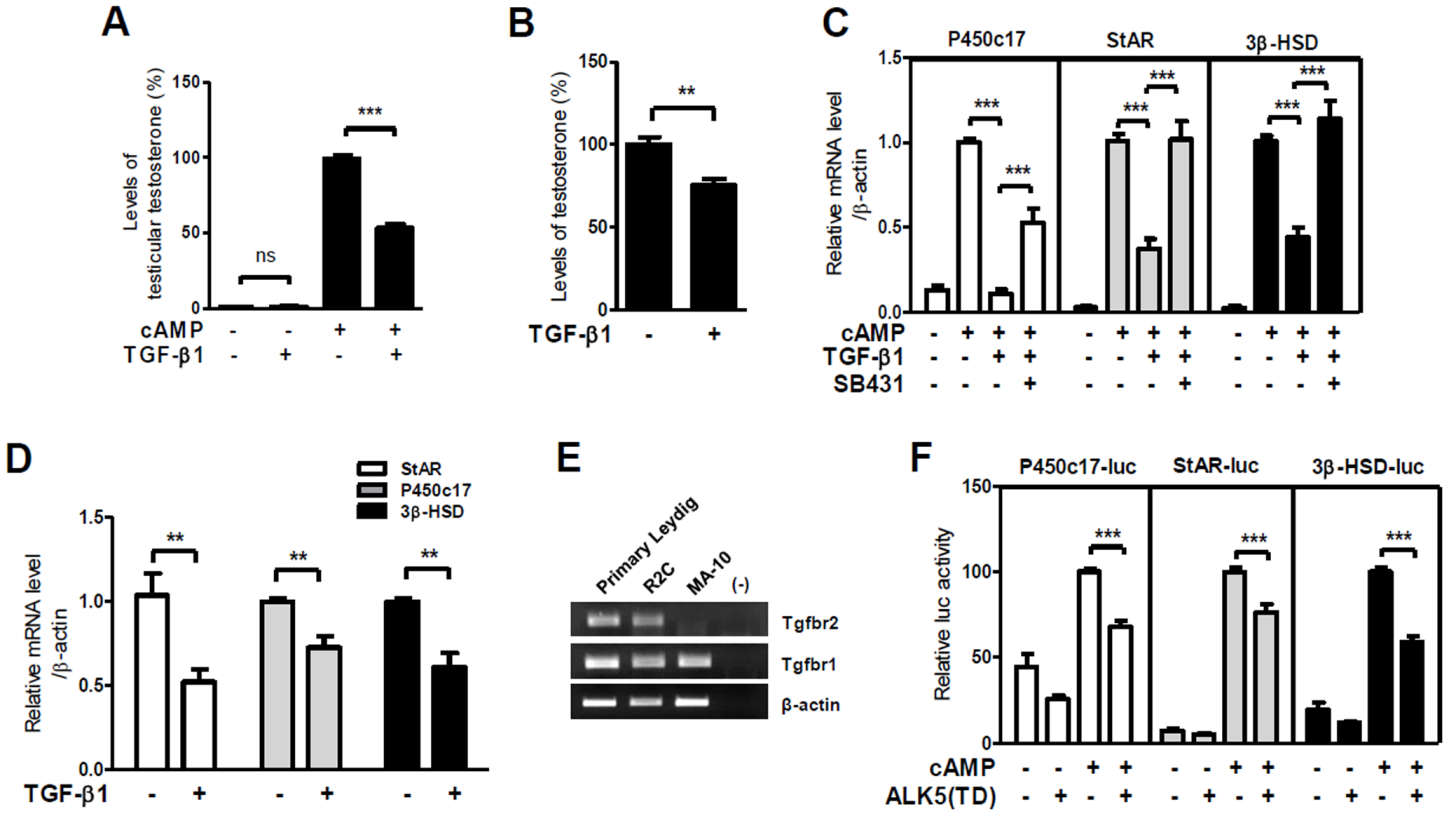
TGF-β1/ALK5 signaling represses cAMP-induced steroidogenic gene expression in Leydig cells. (**A and B**) The culture medium of purified mouse primary Leydig cells treated with 300 µM of 8-Br-cAMP and 5 ng/ml of TGF-β1 (A) and R2C cells treated with vehicle or 5 ng/ml of TGF-β1 (B) for 24 hours was collected for the measurement of testosterone levels by RIA. (**C and D**) The expression levels of steroidogenic genes in primary Leydig cells (C), which were treated with 300 µM of 8-Br-cAMP, 2.5 ng/ml of TGF-β1 and 10 µM SB431542 for 24 hours, and R2C cells (D), which were treated with 5 ng/ml of TGF-β1 for 24 hours, were analyzed by qRT-PCR. (**E**) The expression level of Tgfbr2 and Tgfbr1 was analyzed using total RNAs from primary Leydig, R2C and MA-10 cells by RT-PCR. (**F**) MA-10 cells were transiently transfected with the ALK5 (TD; constitutively active form) expression plasmid, along with an indicated reporter of the natural promoter, in medium containing 5% charcoal stripped FBS. Twenty four hours after transfection, the cells were treated with 300 µM of 8-Br-cAMP for 24 hours and harvested for luciferase assay. The pSV-β-gal expression plasmid was used as a control for transfection efficiency. The data are presented as the mean ± SEM of at least three independent experiments. **, P<0.01; ***, P<0.001; ns, not significant.

Based on a previous report that TGF-β1 inhibits cAMP-induced testosterone formation in Leydig cells [25], we first assessed the effect of TGF-β1 on cAMP-induced steroidogenic gene expression. TGF-β1 treatment repressed cAMP-induced testosterone production in mouse primary Leydig cells (Fig. 1A) and R2C rat Leydig cell line, which is constitutively steroidogenic in nature (Fig. 1B). In addition, TGF-β1 treatment significantly decreased cAMP-induced mRNA levels of steroidogenic genes such as P450c17, StAR and 3β-HSD in primary Leydig cells (Fig. 1C). Similar inhibitory effects of TGF-β1 on steroidogenic gene expression were also observed in R2C cells (Fig. 1D). The inhibitory effect of TGF-β1 on cAMP-induced P450c17, StAR and 3β-HSD gene expression in primary Leydig cells was blocked by treatment with SB431542, a specific inhibitor of TGF-β1 type I receptor ALK5 (Fig. 1C).

We next investigated whether TGF-β1/ALK5 signaling inhibits the expression of steroidogenic genes by affecting their promoter activity using the natural promoter-reporter construct of steroidogenic genes in the mouse Leydig MA-10 cell line. MA-10 cells, which respond to LH/cAMP signals resulting in the upregulation of steroidogenic gene expression, rarely express the TGF-βRII receptor gene (Fig. 1E). Therefore, MA-10 cells were transfected with an expression plasmid for the constitutively active ALK5 mutant, ALK5 (TD; kinase active mutant T204D), which is enough for the activation of TGF-β1 downstream signaling without TGF-βRII expression [27]. The overexpression of ALK5 (TD) significantly repressed the cAMP-induced promoter activity of P450c17, StAR and 3β-HSD genes (Fig. 1F). Together, these data suggest that TGF-β1/ALK5 signaling inhibits the expression of steroidogenic genes by regulating promoter activity.

### TGF-β1/ALK5 signaling inhibits Nur77 transactivation of steroidogenic gene promoters

**Figure 2:**
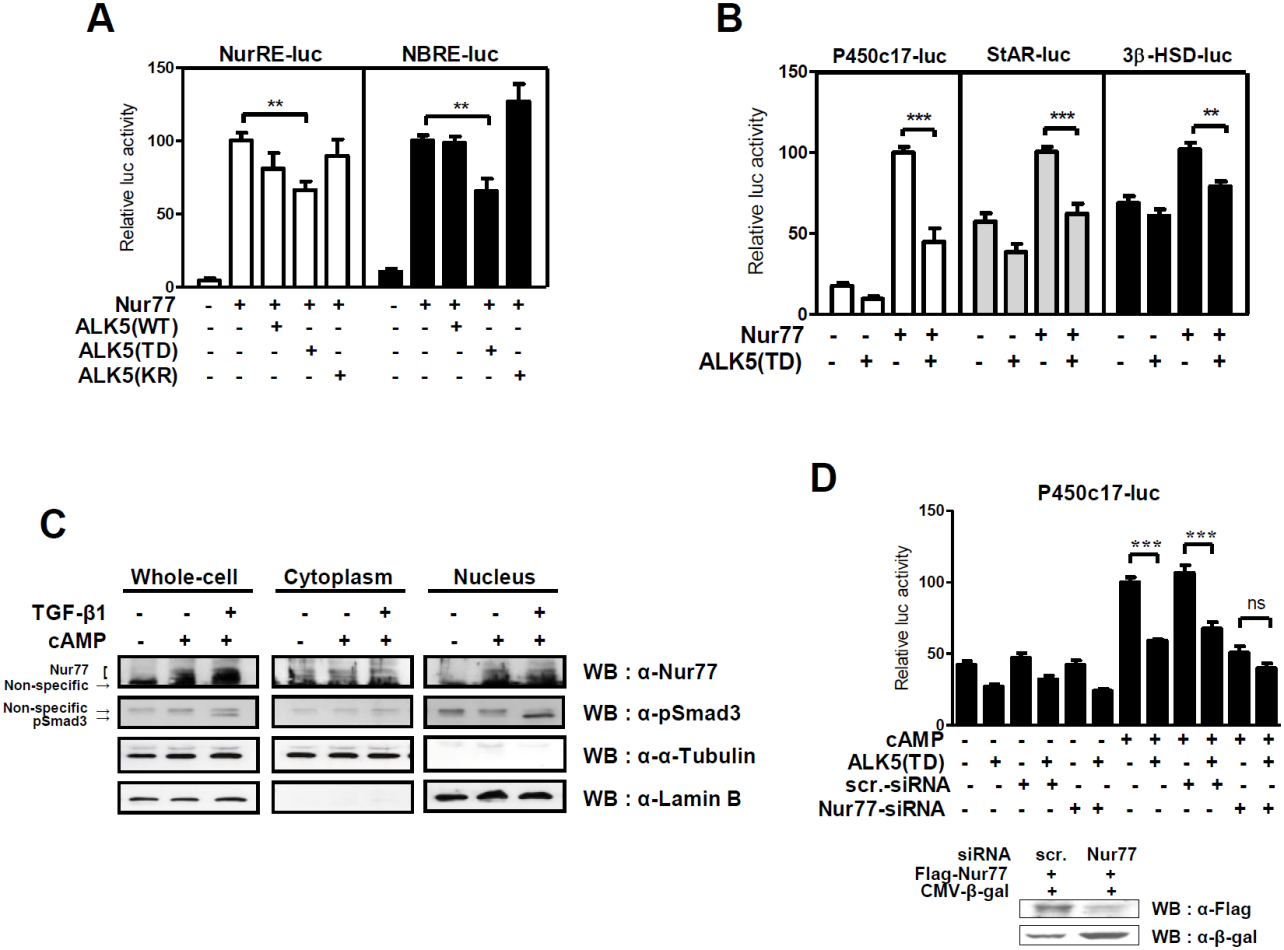
ALK5 signaling inhibits Nur77 transactivation of steroidogenic gene promoters. (**A and B**) MA-10 cells were transiently transfected with the ALK5 WT (wild type), ALK5 mutant (TD; constitutively active form or KR; inactive form), and Nur77 expression plasmids, along with the indicated reporter. The CMVβ expression plasmid was used as a control for transfection efficiency. (**C**) Whole cell extracts and subcellular fractions of primary Leydig cells, which were treated with 300 µM of 8-Br-cAMP and 2.5 ng/ml of TGF-β1 for 4 hours, were analyzed by western blot analysis with anti-Nur77, anti-pSmad3, anti-α-Tubulin (cytoplasmic marker) and anti-Lamin B (nuclear marker) antibodies. (**D**) MA-10 cells were transiently transfected with scrambled or Nur77 siRNA, ALK5 (TD) expression plasmid and P450c17 promoter reporter (top). Silenced Nur77 protein levels in HEK293T cells, which were transiently transfected with scrambled or Nur77 siRNA, Flag-Nur77 and CMVβ expression vector for 48 hours, were determined by western blot analysis (bottom). The data are presented as the mean ± SEM of at least three independent experiments. **, P<0.01; ***, P<0.001; ns, not significant.

The orphan nuclear receptor Nur77 is a major transcription factor that regulates the expression of steroidogenic genes upon cAMP activation in Leydig cells [2], [11], [12]. To investigate the effect of TGF-β1/ALK-5 signaling on Nur77 transactivation, MA-10 cells were transiently transfected with the expression plasmid of ALK5 (WT; wild type) or ALK5 mutants, ALK5 (TD; kinase active mutant T204D) and ALK5 (KR; kinase dead mutant K232R) [27]. The expression of ALK5 (TD), but not ALK5 (WT) and ALK5 (KR), decreased Nur77-induced reporter activity of both NurRE-Luc and NBRE-Luc, which contain the Nur77 binding site (Fig. 2A). The overexpression of ALK5 (TD) also repressed the Nur77-induced promoter activity of the P450c17, StAR and 3β-HSD genes (Fig. 2B). To examine the possibility that TGF-β1 signaling may affect the total levels and/or nuclear localization of Nur77 protein, subcellular analysis was performed with primary Leydig cells which were treated cAMP and TGF-β1. cAMP-induced Nur77 protein levels, both the total and nuclear, were little altered with TGF-β1 treatment (Fig. 2C). These results suggest that TGF-β1/ALK5 signaling represses Nur77 transactivation, with little effect on Nur77 protein levels.

To confirm that TGF-β1/ALK5 signaling distinctly inhibits cAMP-induced steroidogenic gene expression by cross-talk with Nur77, we performed reporter assays using Nur77 small interfering RNA (siRNA). cAMP-induced P450c17 promoter activity was significantly repressed by ALK5 (TD) expression in control cells transfected with scrambled siRNA, but ALK5 (TD)-mediated repression was not observed in Nur77 knockdown cells transfected with Nur77 siRNA (Fig. 2D). Taken together, these results suggest that TGF-β1/ALK5 signaling represses cAMP-induced steroidogenic gene expression mainly through cross-talk with Nur77, inhibiting Nur77 transactivation.

### ALK5-activated Smad3 represses Nur77 transactivation

**Figure 3:**
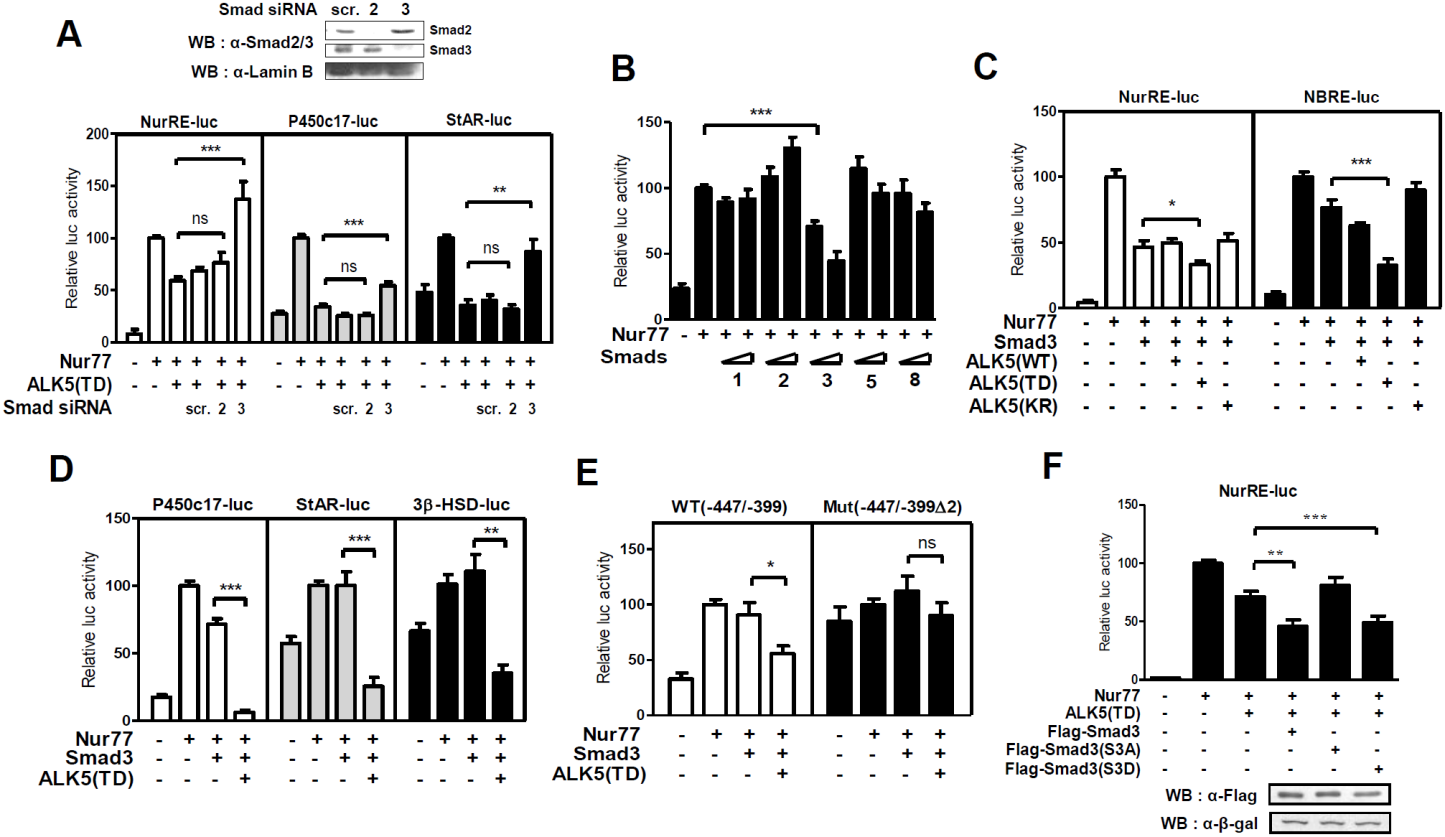
ALK5-activated Smad3 represses Nur77 transactivation of steroidogenic gene promoters. (**A**) MA-10 cells were transiently transfected with siRNA, Nur77, ALK5 (TD) and an indicated reporter for 48 hours and were harvested for luciferase assay. The CMVβ expression plasmid was used as a control for transfection efficiency (bottom). The silencing efficiencies of Smad2 and Smad3 siRNA were determined by western blot analysis (top). (**B**) MA-10 cells were transiently transfected with Nur77, increasing amounts of Smad (60 and 150 ng) expression plasmids and the NBRE reporter construct. (**C–E**) MA-10 cells were transiently transfected with ALK5 WT, ALK5 mutant (TD or KR), Smad3 and Nur77 expression plasmids, along with the indicated reporter construct. (**F**) MA-10 cells were transiently transfected with expression plasmids of Nur77, ALK5 (TD), Flag-Smad3 (WT) or a phosphorylation mutant (S3A or S3D), and NurRE-luc reporter construct (top). A similar amount of expressed protein was confirmed by western blot analysis (bottom). The data are presented as the mean ± SEM of at least three independent experiments. *, P<0.5; **, P<0.01; ***, P<0.001; ns, not significant.

ALK5 activates Smad2 and Smad3 as downstream effectors of TGF-β1 signaling [17]. To verify whether Smad2 and Smad3 are involved in the repression of Nur77 transactivation by TGF-β1/ALK5 signaling, we depleted endogenous Smad2 or Smad3 in MA-10 cells by utilizing siRNA, which resulted in a substantial reduction in Smad2 or Smad3 protein level (Fig. 3A top). AKL5 (TD) expression decreased the Nur77-induced promoter activity of NurRE and the steroidogenic genes P450c17 and StAR. Smad3 knockdown with Smad3 siRNA was associated with the recovery of promoter activity, whereas Smad2 knockdown with Smad2 siRNA, as well as the control transfected with scrambled siRNA, had no significant effect (Fig. 3A bottom). Moreover, the overexpression of Smad3, but not Smad2 and other receptor-regulated Smads such as Smad1, Smad5 and Smad8, distinctly repressed Nur77 transactivation (Fig. 3B).

The overexpression of Smad3 decreased Nur77 transactivation with NurRE-luc and NBRE-luc reporters. The coexpression of Smad3 with ALK5 (TD), but not ALK5 (WT) and ALK5 (KR), further inhibited Nur77 transactivation (Fig. 3C), suggesting that ALK5-activated Smad3 represses Nur77 transactivation. We further confirmed the inhibitory effect of activated Smad3 on Nur77 transactivation of steroidogenic gene promoters. As expected, the coexpression of ALK5 (TD) and Smad3 strongly repressed Nur77-induced P450c17, StAR and 3β-HSD promoter activity (Fig. 3D). Furthermore, the promoter activity of WT(−447/−399) P450c17-Luc containing the Nur77 binding site, but not Mut(−447/−399Δ2) P450c17-Luc, in which the Nur77 binding site was mutated, was decreased by the coexpression of ALK5 (TD) and Smad3 (Fig. 3E). Interestingly, Smad3 overexpression itself strongly repressed the activity of NurRE reporter, in which Nur77 binds as dimer, in comparison with NBRE reporter and NBRE-containing P450c17, StAR and 3β-HSD promoters, in which Nur77 binds as monomer.

ALK5 activated by TGF-β1 phosphorylates the C-terminal Ser-Ser-X-Ser (SSXS) motifs of Smad3. Smad3 subsequently translocates into the nucleus to act as a transcription regulator [17]. To confirm that TGF-β1-induced phosphorylation of Smad3 is necessary for the repression of Nur77 transactivation, we utilized two Smad3 mutants, the phosphorylation-mimic form Smad3 (S3D) and the phosphorylation-defective form Smad3 (S3A). In the presence of activated ALK5 signaling, the overexpression of the wild-type Smad3 and the Smad3 (S3D) mutant, but not Smad3 (S3A), repressed Nur77 transactivation (Fig. 3F), suggesting a requirement of ALK5-induced phosphorylation of Smad3 for the repression of Nur77 transactivation. Taken together, these results suggest that ALK5-activated Smad3, downstream of TGF-β1 signaling, inhibits Nur77 transactivation.

### Smad3 physically interacts with Nur77

**Figure 4:**
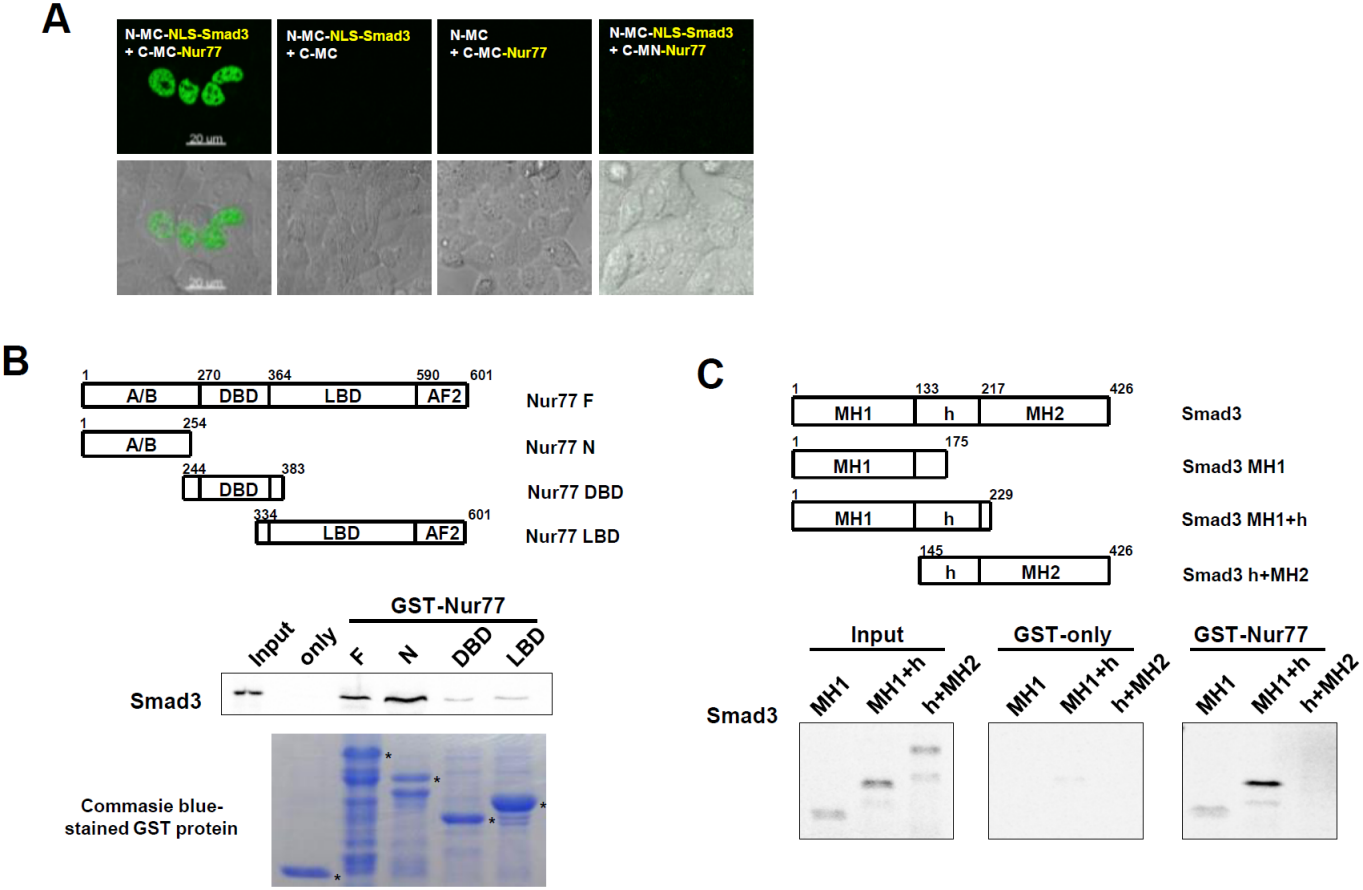
Smad3 physically interacts with Nur77. (**A**) mKG_N-MC-NLS-Smad3 and mKG_C-MC-Nur77 were transfected into HeLa cells for 24 hours. Interaction between Nur77 and NLS-Smad3 yielded fluorescent green signals in the nucleus. The single fusion protein alone (mKG_N-MC-NLS-Smad3 or mKG_C-MC-Nur77) and another pair (mKG_N-MC-NLS-Smad3 and mKG_C-MN-Nur77) gave no fluorescent signal. The scale bars represent 25 µm. (**B**) [^35^S] methionine-labeled Smad3 produced by *in vitro* translation was incubated with the GST-Nur77 fusion protein and its deletion mutants. Coomassie blue staining shows the protein level of the purified GST, GST-Nur77 and GST-Nur77 deletion mutant (bottom). (**C**) [^35^S] methionine-labeled Smad3 deletion mutants were incubated with GST-Nur77 fusion protein. The data are representative of three independent experiments.

To investigate whether functional interaction between Smad3 and Nur77 involves their physical association, protein fragment complementation analysis was performed [37]. In HeLa cells, the combination of the Nur77 fusion protein (mKG_C-MC-Nur77) and the NLS (nuclear localization signal) tagged-Smad3 fusion protein (mKG_N-MC-NLS-Smad3), which is constitutively localized in the nucleus, yielded strong fluorescent signals in the nucleus, suggesting their interaction in the nucleus (Fig. 4A). This is consistent with the fact that TGF-β1-activated Smad3 and Nur77 are subcellularly distributed in the nucleus.

To determine which regions of Nur77 and Smad3 are responsible for their interaction, GST pull-down assays were performed. [^35^S] methionine-labeled Smad3 was incubated with the GST-fusion protein of full-length Nur77 or deleted mutants. Smad3 strongly interacted with both Nur77 full-length and Nur77 N mutant, but very weakly interacted with Nur77 DBD and LBD mutants (Fig. 4B). When [^35^S] methionine-labeled Smad3 deleted mutants were incubated with GST-fusion protein of full-length Nur77, Nur77 interacted with Smad3 MH1 and MH1+h mutants, but not the h+MH2 mutant (Fig. 4C). Taken together, these results suggest that Smad3 directly interacts with Nur77 and that the N-terminal region of Nur77 and the MH1 and hinge regions of Smad3 are mainly responsible for their interaction.

### TGF-β1 signaling inhibits the recruitment of Nur77 to DNA

**Figure 5:**
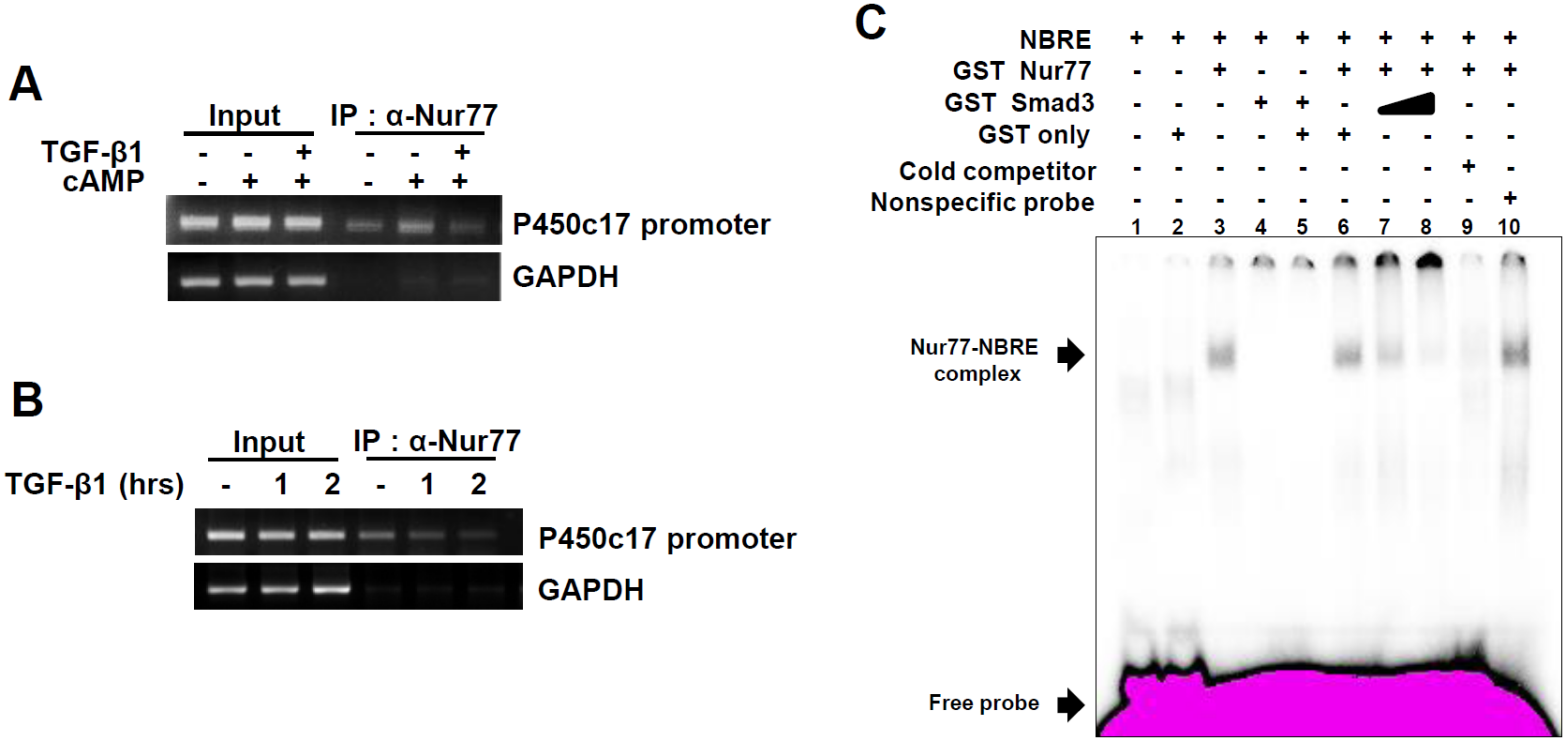
TGF-β1 signaling interferes with Nur77 binding to DNA. (**A and B**) TGF-β1 inhibits the recruitment of Nur77 to the P450c17 promoter. ChIP assays were performed using purified primary Leydig cells treated with 300 µM of 8-Br-cAMP and 10 ng/ml of TGF-β1 for 2 hours (A) and R2C cells treated with 10 ng/ml of TGF-β1 for the indicated time (B). Anti-Nur77 antibody was used for immunoprecipitation. The immunoprecipitates were analyzed by PCR using a pair of specific primers spanning a region containing the Nur77 binding site of the P450c17 promoter. A negative control PCR for nonspecific immunoprecipitation was performed using primers specific to the GAPDH coding region. (**C**) The interference with Nur77 binding to NBRE by Smad3. The GST-Nur77 fusion protein was incubated with α-^32^P-labeled NBRE oligonucleotide, along with increasing amounts of purified GST-Smad3 (lanes 7 and 8) proteins. A 100-fold excess of cold NBRE oligomer (lane 9) or nonspecific oligomer (ARE, lane 10) was added. Positions of the specific protein-DNA complex and the free probe are indicated. The data are representative of three independent experiments.

Because TGF-β1 signaling repressed Nur77 transactivation at the natural promoter of steroidogenic genes (Fig. 3D), we performed ChIP assays to investigate whether TGF-β1 signaling affects the recruitment of Nur77 to DNA. Following the induction of Nur77 expression in mouse primary Leydig cells by cAMP treatment, the association of Nur77 with the endogenous P450c17 promoter increased. However, TGF-β1 co-treatment with cAMP resulted in decreased Nur77 recruitment to the P450c17 promoter (Fig. 5A). Moreover, TGF-β1 treatment of R2C cells also inhibited Nur77 recruitment to the endogenous P450c17 promoter in a time-dependent manner (Fig. 5B). These results indicate that TGF-β1 signaling inhibits Nur77 recruitment to DNA.

To further confirm that Smad3 affects the DNA binding activity of Nur77, electrophoretic mobility shift assays (EMSAs) were performed using the α-^32^P-labeled NBRE oligonucleotide, GST-Nur77, and the GST-Smad3 fusion protein (Fig. 5C). α-^32^P-labeled NBRE oligonucleotide formed a complex with GST-Nur77 protein (lane 3), which was reduced by a 100-fold excess of cold NBRE oligomer (lane 9), but not by nonspecific oligomer (lane 10), indicating the formation of a specific NBRE/GST-Nur77 complex. Coincubation of GST-Smad3 with GST-Nur77, along with α-^32^P-labeled NBRE oligonucleotide, eliminated the NBRE/GST-Nur77 complex in a dose-dependent manner (lanes 7 and 8), while coincubation of GST-Smad3 with GST only did not (lanes 6). Taken together, these results imply that the Smad3 activated by TGF-β1 signaling interacts with Nur77 and then inhibits Nur77 recruitment to target promoters through interfering with its DNA binding activity, which results in the repression of Nur77 transactivation.

### Knock-out of TGF-β type II receptor (Tgfbr2) in Leydig cells decreases the inhibitory effects of TGF-β1 on steroidogenesis

**Figure 6:**
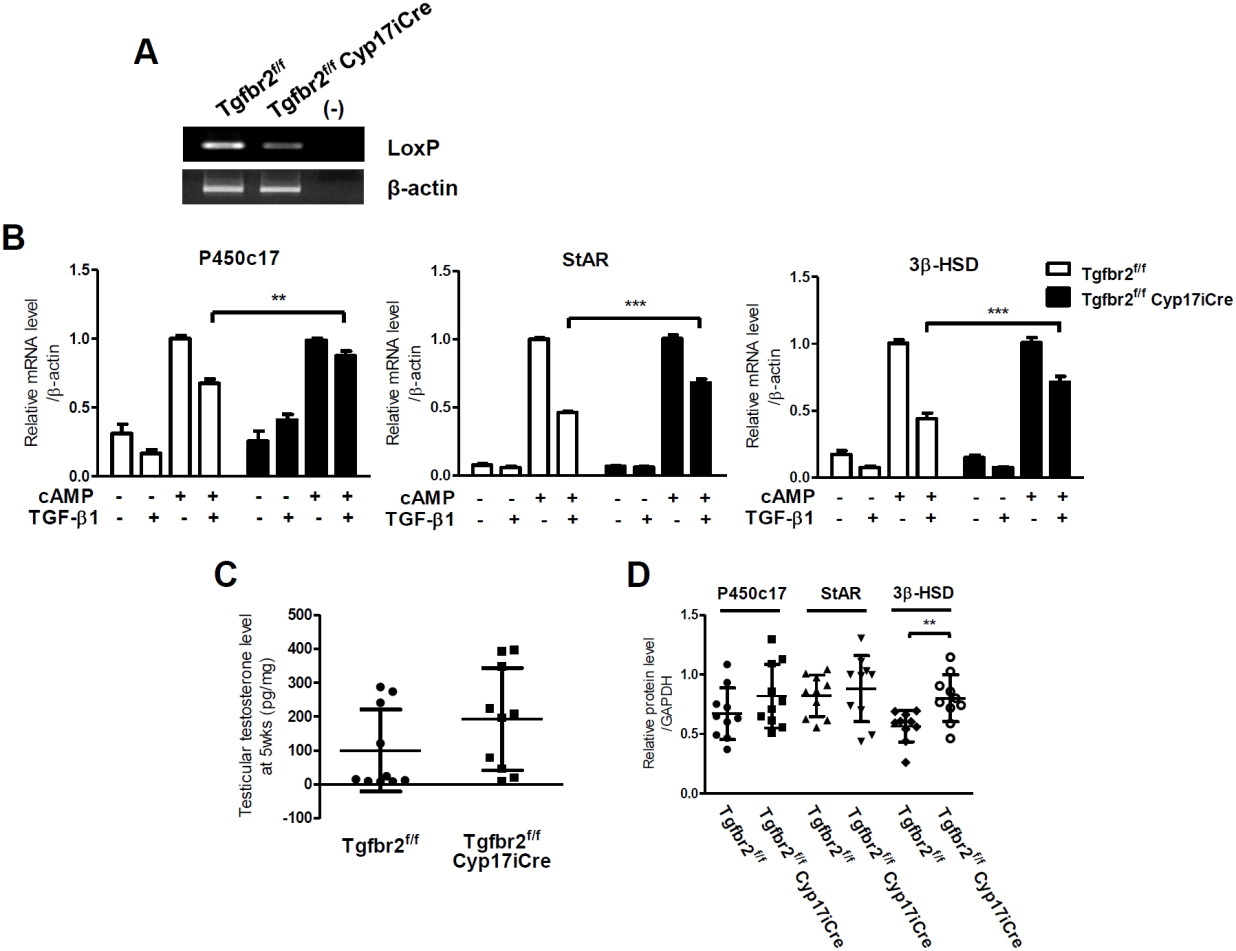
TGF-β1 signaling regulates steroidogenic gene expression, affecting testicular testosterone levels in mice. (**A**) Decreased Tgfbr2^fl^°^x^ allele in purified primary Leydig cells isolated from mice harboring the Cyp17iCre transgene. The genomic DNA isolated from primary Leydig cells of Tgfbr2^flox/flox^ and Tgfbr2^flox/flox^ Cyp17iCre mice was amplified for Tgfbr2 intron region containing the LoxP site. A pair of β-actin primers was used as the control for the amount of genomic DNA. (**B**) Decreased TGF-β1-mediated repression of steroidogenic gene expression with Tgfbr2 silencing. Purified primary Leydig cells from the testes of 12-week-old Tgfbr2^flox/flox^ (n = 6) and Tgfbr2^flox/flox^ Cyp17iCre (n = 6) mice were treated with 300 µM of 8-Br-cAMP and 2 ng/ml of TGF-β1 for 24 hours, and mRNA expression levels were measured using qRT-PCR. β-actin expression was used as a loading control. The data are presented as the mean ± SEM. **, P<0.01; ***, P<0.01. (**C**) Testicular testosterone levels were measured by RIA in the testes of 5 week-old Tgfbr2^flox/flox^ and Tgfbr2^flox/flox^ Cyp17iCre mice. (**D**) Total protein (100 µg) from the testes of 5 week-old Tgfbr2^flox/flox^ and Tgfbr2^flox/flox^ Cyp17iCre mice was subjected to western blot analysis for protein levels of steroidogenic genes. The relative level of each protein/GAPDH was quantified by densitometric analysis using Image J software. In panels C and D, the data are presented as the mean ± SD (n = 10). **, P<0.01.

To explore the effect of TGF-β1 signaling on testicular steroidogenesis in animals, we established Leydig cell-specific TGF-β type II receptor (Tgfbr2) conditional knock-out mice, Tgfbr2^flox/flox^ Cyp17iCre. Because receptor type II is specific for TGF-β, unlike receptor type I, the deletion of TGF-β receptor type II disturbs only TGF-β signaling, and has no effect on the signaling of the other subfamily members. Unfortunately, the knock-out efficiency for the Tgfbr2^fl^°^x^ allele in Leydig cells of Tgfbr2^flox/flox^ Cyp17iCre male mice was moderate (Fig. 6A), and no differences in phenotype during testes development was observed between Tgfbr2^flox/flox^ Cyp17iCre and Tgfbr2^flox/flox^ mice. Nevertheless, in primary Leydig cells isolated from Tgfbr2^flox/flox^ Cyp17iCre male mice, TGF-β1 treatment repressed the cAMP-induced expression of P450c17, StAR and 3β-HSD significantly less than in primary Leydig cells isolated from the control Tgfbr2^flox/flox^ male mice (Fig. 6B). Moreover, Tgfbr2^flox/flox^ Cyp17iCre male mice showed a tendency towards an increase in testicular testosterone level when compared with Tgfbr2^flox/flox^ male mice (Fig. 6C). In addition, the protein level of 3β-HSD in the testes of Tgfbr2^flox/flox^ Cyp17iCre mice was significantly higher than the level in the testes of Tgfbr2^flox/flox^ mice, although the protein level of P450c17 and StAR showed only a tendency to increase (Fig. 6D). These results suggest that TGF-β1 signaling down-regulates testicular steroidogenesis, at least in part, by inhibiting the expression of steroidogenic genes in vivo.

## Discussion

Several studies have demonstrated that TGF-β signaling is involved in testicular steroidogenesis, decreasing LH/hCG receptor expression [23], [38]. However, TGF-β1 treatment of primary Leydig cells reduced the production of cAMP-induced testosterone as well as hCG-stimulated testosterone [25], [38]. This TGF-β1 effect on cAMP-induced steroidogenesis indicates that TGF-β1 may also act downstream of LH/cAMP signaling to control steroidogenesis, suggesting the possibility that TGF-β1 inhibits steroidogenesis through regulating the expression of cAMP-induced steroidogenic genes. In the present study, we show that TGF-β1 signaling represses the expression of steroidogenic genes via cross-talk with Nur77 transcription factor, resulting in the reduction of testosterone production in Leydig cells.

SF-1, LRH-1 and Nur77 are the primary transcription factors that regulate testicular steroidogenesis [39]. These transcription factors share a similar DNA binding sequence [40]. Unlike SF-1 and LRH-1, Nur77 is rapidly and strongly induced in response to hormonal stimulation, including LH/cAMP, in Leydig cells [41]. Moreover, it is still not fully understood how the association of SF-1 and LRH-1 with the promoter of steroidogenic genes is increased following cAMP stimulation, even though its protein levels remain unchanged [42], [43]. In this study, active ALK5 expression repressed both basal and cAMP-induced activity of the P450c17 promoter in MA-10 cells (Fig. 2D), which express high basal levels of SF-1 and LRH-1, but not Nur77 [33], [41]. In addition, ALK5-activated Smad3 repressed the transcriptional activity of SF-1 and LRH-1 as well as Nur77 (data not shown). Therefore, the repression of the basal promoter activity of P450c17 with active ALK5 expression may be related to the inhibition of SF-1 and LRH-1 transactivation by ALK5 signaling. Consistent with this speculation, Smad3 has been reported to inhibit SF-1-induced P450c17 promoter activity in adrenocortical cells [44].

In most cell types, TGF-β1 binds to the ubiquitously expressed ALK5 receptor that activates Smad2 and Smad3. However, recent studies have revealed that TGF-β1 also mediates the activation of Smad1 and Smad5 via the ALK1 receptor in endothelial cells [45]. It has been suggested that ALK1 activation triggers cell proliferation and migration, whereas ALK5 activation has the opposite effect in endothelial cells [46]. Leydig cells express ALK1 and its downstream effector Smad1/5, as well as ALK5 and Smad2/3 [47], [48]. In humans, the ALK1 immunostaining signal is stronger in testes with Leydig cell hyperphasia than in testes with Sertoli cell-only syndrome or hypospermatogenesis [48]. Moreover, the decreased expression of ALK1 by aging or hCG treatment in Leydig cells suggests that TGF-β1 signaling through ALK1 is involved in the proliferation of Leydig cells [47]. In this study, we concentrated on the regulation of Nur77 transactivation by ALK5/Smad3 signaling because the overexpression of other receptor-regulated Smads such as Smad1, Smad2, Smad5 and Smad8, showed little effect on Nur77 transactivation (Fig. 3B). Nevertheless, further studies are necessary to elucidate whether TGF-β1/ALK1 signaling is also related to testicular steroidogenesis.

Numerous studies have shown that Smad3 represses gene transcription through direct binding to DNA or through association with other transcription factors. For example, Smad3 represses the transcription of coxsackie and adenovirus receptor (CAR) [49] and insulin [50] by directly binding to the Smad-binding elements (SBEs) in their promoters. On the other hand, Smad3 has also been shown to repress gene transcription by inhibiting the transactivation of MyoD [51], Pax6 [52] and AR [53], by interfering with the binding of the transcription factors to DNA. In the present study, Smad3 repressed transcription of steroidogenic genes by inhibiting Nur77 transactivation through interfering with Nur77 binding to DNA (Fig. 5), therefore extending the list of indirect target genes of Smad3.

Tgfbr2^flox/flox^ Cyp17iCre male mice, which are specifically deleted for the TGF-β receptor type II gene in testicular Leydig cells, showed a tendency towards an increase in testicular testosterone levels and steroidogenic gene expression compared with Tgfbr2^flox/flox^ male mice (Fig. 6C and D). In purified primary Leydig cells isolated from Tgfbr2^flox/flox^ Cyp17iCre male mice, however, TGF-β1-mediated repression of cAMP-induced P450c17, StAR and 3β-HSD expression was significantly less compared with Tgfbr2^flox/flox^ male mice (Fig. 6B). It has been reported that TGF-β1 null male mice have diminished serum and intratesticular testosterone due to disrupted pituitary gonadotropin secretion [24]. Given that TGF-β1 signaling functions in the hypothalamic-pituitary-gonadal axis as well as intratesticular testosterone synthesis, the dysregulation of intratesticular testosterone synthesis due to Leydig cell-specific deficiency of TGF-β1 signaling in Tgfbr2^flox/flox^ Cyp17iCre testes might be partially restored by TGF-β1 signaling in the brain. This model may explain our observation of significant differences in steroidogenic gene expression between Tgfbr2^flox/flox^ Cyp17iCre and Tgfbr2^flox/flox^ male mice in the purified Leydig cells, but not in whole testes.

In the present study, we investigated whether TGF-β1 inhibits the expression of steroidogenic genes in Leydig cells to control testicular steroidogenesis and the molecular mechanisms involved in such regulation. We demonstrate that ALK5-activated Smad3 inhibits the transactivation of Nur77 through the inhibition of Nur77 binding to DNA, resulting in decreased expression of steroidogenic genes. The inhibitory effects of TGF-β1, which were also observed in primary Leydig cells isolated from Tgfbr2^flox/flox^ Cyp17iCre male mice, supports a negative role for TGF-β1 in testicular steroidogenesis. Together, these findings may provide a mechanical understanding for the local regulation of testicular steroidogenesis by TGF-β1/ALK5/Smad3 signaling.
